# T cells expressing CD19-specific Engager Molecules for the Immunotherapy of CD19-positive Malignancies

**DOI:** 10.1038/srep27130

**Published:** 2016-06-03

**Authors:** Mireya Paulina Velasquez, David Torres, Kota Iwahori, Sunitha Kakarla, Caroline Arber, Tania Rodriguez-Cruz, Arpad Szoor, Challice L. Bonifant, Claudia Gerken, Laurence J. N. Cooper, Xiao-Tong Song, Stephen Gottschalk

**Affiliations:** 1 Center for Cell and Gene Therapy, Texas Children’s Hospital, Houston Methodist Hospital, Baylor College of Medicine, Houston, Texas 77030, USA; 2 Texas Children’s Cancer Center, Texas Children’s Hospital, Baylor College of Medicine, Houston, Texas 77030, USA; 3 Department of Pediatrics, Baylor College of Medicine, Houston, Texas 77030, USA; 4 Division of Pediatrics, The University of Texas MD Anderson Cancer Center, Houston, TX, USA; 5 Department of Pathology and Immunology, Baylor College of Medicine, Houston, Texas 77030, USA.

## Abstract

T cells expressing chimeric antigen receptors (CARs) or the infusion of bispecific T-cell engagers (BITEs) have shown antitumor activity in humans for CD19-positive malignancies. While BITEs redirect the large reservoir of resident T cells to tumors, CAR T cells rely on significant *in vivo* expansion to exert antitumor activity. We have shown that it is feasible to modify T cells to secrete solid tumor antigen-specific BITEs, enabling T cells to redirect resident T cells to tumor cells. To adapt this approach to CD19-positive malignancies we now generated T cells expressing secretable, CD19-specific BITEs (CD19-ENG T cells). CD19-ENG T cells recognized tumor cells in an antigen-dependent manner as judged by cytokine production and tumor killing, and redirected bystander T cells to tumor cells. Infusion of CD19-ENG T cells resulted in regression of leukemia or lymphoma in xenograft models and a survival advantage in comparison to control mice. Genetically modified T cells expressing engager molecules may present a promising addition to current CD19-targeted immunotherapies.

The treatment of CD19-positive hematological malignancies including acute lymphoblastic leukemia (ALL) and Non-Hodgkin Lymphoma (NHL) has made great strides in the last decades^1^,^2^,^3^,^4^. However, current treatment regimens are associated with significant acute and long-term toxicities^5^. In addition, patients with recurrent or chemotherapy refractory disease have a poor prognosis^6^, highlighting the need to develop new therapeutic approaches that improve outcomes and reduce treatment-related complications for all patients.

Promising immunotherapy approaches for CD19-positive hematological malignancies include the adoptive transfer of T cells that are genetically modified to express CD19-specific chimeric antigens receptors (CARs) or the infusion of bispecific antibodies that redirect resident T cells to CD19^7^,^8^,^9^,^10^,^11^,^12^,^13^,^14^,^15^. The most successful bispecific antibodies in clinical studies are bispecific T-cell engagers (BITEs), which consist of 2 single chain variable fragments (scFVs) connected by a short linker^15^. While the CD19-specific BITE blinatumomab received FDA approval in 2014^16^,^17^, BITEs have a short half-life, requiring continuous infusion that may be associated with toxicities, lack active biodistribution, and inability to self-amplify^18^,^19^. One potential strategy to overcome these limitations is the genetic modification and adoptive transfer of T cells that secrete diabodies^20^ or T-cell engagers (ENG T cells)^21^, since T cells can actively secrete molecules at tumor sites, and persist for several weeks post infusion. While ENG T cells have been explored in preclinical models for solid tumors^21^, no data is currently available for hematological malignancies. In this study, we characterize ENG T cells specific for CD19-positive malignancies (CD19-ENG T cells) and show that they are activated and kill tumor cells in an antigen dependent manner, are able to recruit bystander T cells to tumor cells, and have antitumor activity in preclinical models.

## Materials and Methods

### Cell lines and culture conditions

The Ph-positive acute B lymphoblastic leukemia (ALL) cell line BV173 (German Collection of Microoganisms and Cell Cultures, Braunschweig, Germany) and Burkitt’s lymphoma cell lines Daudi and Raji (ATCC, Manassas, VA) were used as CD19-positive targets. The generation of firefly luciferase (ffLuc)-expressing BV173 (BV173.ffLuc) and Daudi (Daudi.ffLuc) cells were described previously^22^,^23^. K562 (chronic myelogenous leukemia, ATCC) and A549 (lung carcinoma, ATCC) cell lines were used as negative controls. All cell lines were grown in RPMI 1640 (Thermo Scientific). 293T cells (ATCC) were used for packaging retroviral vectors and grown in DMEM. All media was supplemented with 10–20% FBS (Thermo Scientific) and 2 mmol/L GlutaMAX-I (Invitrogen, Carlsbad, CA).

### Construction of retroviral vectors encoding T-cell enganger molecules

The construction of the CD19-specific engager molecule has been previously reported^21^. Briefly, a mini gene encoding a CD19-specific engager molecule containing the immunoglobulin heavy-chain leader peptide, the CD19-specific scFv (FMC63)^24^, a short serine-glycine linker, and a CD3-specific scFV derived from OKT3 was synthesized by Invitrogen (Carlsbad, CA) and subcloned into pSFG-IRES-mOrange (provided by Dr. Vera, Baylor College of Medicine). The retroviral vector encoding the EphA2-specific T-cell engager was generated in a similar fashion using the EphA2-specific scFv 4H5^25^. RD114-pseudotyped retroviral particles were generated as previously described^26^.

### Generation of Engager T cells

All methods involving human subjects were carried out in accordance to the Declaration of Helsinki. Human peripheral blood mononuclear cells (PBMCs) from healthy donor were obtained under a Baylor College of Medicine IRB approved protocol, after acquiring informed consent. PBMCs were stimulated on OKT3 (1 μg/mL, CRL-8001, ATCC) and CD28 (1 μg/mL, BD Bioscience) antibodies-coated non-tissue culture treated 24-well plates. Human interleukin 2 (IL2) (200 U/mL, Biological Research Branch, National Cancer Institute, Frederick, MD) was added to cultures on day 2, and on day 3 T cells were transduced with retroviral particles on RetroNectin (Clontech) coated plates in the presence IL2 (100 U/mL). T cells were subsequently expanded with IL2. Non-transduced (NT) T cells were activated with OKT3/CD28 and expanded in parallel with IL2. Cells were cultured for 7–10 days prior to being used for *in vitro* or *in vivo* experiments.

### Flow cytometric analysis

The expression of mOrange was detected by FACS analysis. For immunophenotyping, cells were stained with CD3-PerCP, CD4-FITC, and CD8-FITC monoclonal antibodies (BD Biosciences, Mountain View, CA). Isotype controls were IgG1-FITC and IgG1-PerCP (both Becton Dickinson, Mountain View, CA). CD19-specific T-cell engagers were detected using a CD19 scFv-specific ID antibody^27^ and an APC Goat antimouse IgG antibody (BD Biosciences)^27^. For each sample, 20,000 cells were analyzed by a FACSCalibur instrument (BD Biosciences) using Cell Quest Software (BD Biosciences).

### Bioassay

NT T cells and Raji cells were plated in a 96 well plate at a 1:1 ratio (1 × 10^5^/well) in 100 μl of complete RPMI. Next 100 μl of serially diluted recombinant CD19xCD3-bispecific engager protein (GenScript, Piscataway, NJ) or conditioned media (obtained from ENG T cells cultured for 24 hour at a concentration of 1 × 10^6^/mL) were added. The 96 well plates were incubated for 14 to 16 hours prior to determining interferon (IFN)γ using an ELISA kit as per the manufacturer’s instructions (R&D Systems, Minneapolis, MN). To determine the ENG concentration in conditioned media a standard curve was constructed using the IFNγ values obtained with serially diluted recombinant CD19-/CD3-specific engager protein.

### Coculture assays

CD19-ENG and EphA2-ENG T cells were plated at a 2:1 ratio with CD19-positive (Daudi, Raji, BV173) or -negative (K562) target cells. Coculture supernatant was collected after 48 hours and used for cytokine analysis. IFNγ and IL2 concentrations were determined using ELISA kits (R&D Systems, Minneapolis, MN) according to the manufacturer’s instructions.

### Cytotoxicity assay

Cytotoxic activity of ENG T cells against targets was determined by standard ^51^Cr release assay. 1 × 10^6^ target cells were labeled with 50μCi ^51^Cr and incubated for 1 hour. Targets were then washed and 5 × 10^3^ cells were cocultured with effector T cells at different effector to target (E:T) ratios. Supernatants were analyzed with a Packard Cobra Quantum gamma counter Model E 5010 (Perkin Elmer, Shelton CT) reader after 4 hour incubation. Lysis was calculated as previously described^21^.

### Transwell Assay

To show that CD19-ENG T cells were able to recruit bystander (non-transduced) T cells we performed transwell assays with GFP.ffLuc-expressing target cells using inserts (6.5 mm in diameter, 0.4 μm pore, polycarbonate, Corning, Corning, NY), which allow for small molecule diffusion but not cell migration. Luciferase activity was determined according to the manufacturer’s instructions using a luciferase assay kit (Promega, Madison, WI) and a Monolight 3010 luminometer (BD Biosciences).

### *In vivo* experiments

Animal experiments were performed on a protocol approved by the Baylor College of Medicine Institutional Animal Care and Use Committee in accordance to the American Association for Laboratory Animal Science. Eight to 10 week old NSG mice (NOD.Cg-Prkdcscid/Il2rgtm1Wjl/SzJ; JAX Mice, Bar Harbor, ME) were sublethally irradiated with 120 cGy 24 hours before the intravenous (i.v.) injection of 3 × 10^6^ BV173.ffLuc cells. Mice were treated i.v. with 1 × 10^7^ CD19-ENG or EphA2-ENG T cells on days 7, 14, and 21 after tumor cell injection. Cell count of infused cells was based on total cell number. Untreated animals served as controls. As indicated, animals received one intraperitoneal (i.p.) dose of 1,500 units IL2 (Biological Research Branch, National Cancer Institute, Frederick, MD) on the day of the T-cell injections. At indicated time points blood was collected by retro-orbital bleeding. Serum cytokine concentrations were determined using Milliplex MAP High Sensitivity Human Cytokine Panel – Premixed 13 Plex (EMD Millipore, Billerica, MA) as per manufacturer’s instructions. In the systemic Daudi model, mice received i.v. 2 × 10^6^ Daudi.ffLuc cells followed by 1 × 10^7^ i.v. T-cell injections on days 3, 6 and 9. In the local Daudi model, mice were injected i.p. with 2.5 × 10^6^ Daudi cells in the right lower quadrant, and 14 days later received i.v. 3 × 10^6^ T-cells CD19-ENG or EphA2-ENG T-cells that were also genetically modified to express eGFP.ffLuc. Mice were imaged using the IVIS® system (IVIS, Xenogen Corp., Alameda, CA) as previously described^22^, and euthanized at predefined endpoints or when they met euthanasia criteria in accordance with the Center for Comparative Medicine at Baylor College of Medicine.

### Statistical analysis

GraphPad Prism 5 software (GraphPad software, Inc.) was used for statistical analysis. Measurement data were presented as mean ± standard deviation (SD). For comparison between two groups, two-tailed t-test was used. For comparisons of three or more groups, the values were analysed by one-way ANOVA with Bonferroni’s post-test. For the mouse experiments, survival, determined from the time of tumor cell injection, was analysed by the Kaplan–Meier method and by the log-rank test.

## Results

### Generation of CD19-ENG T cells

**Figure 1:**
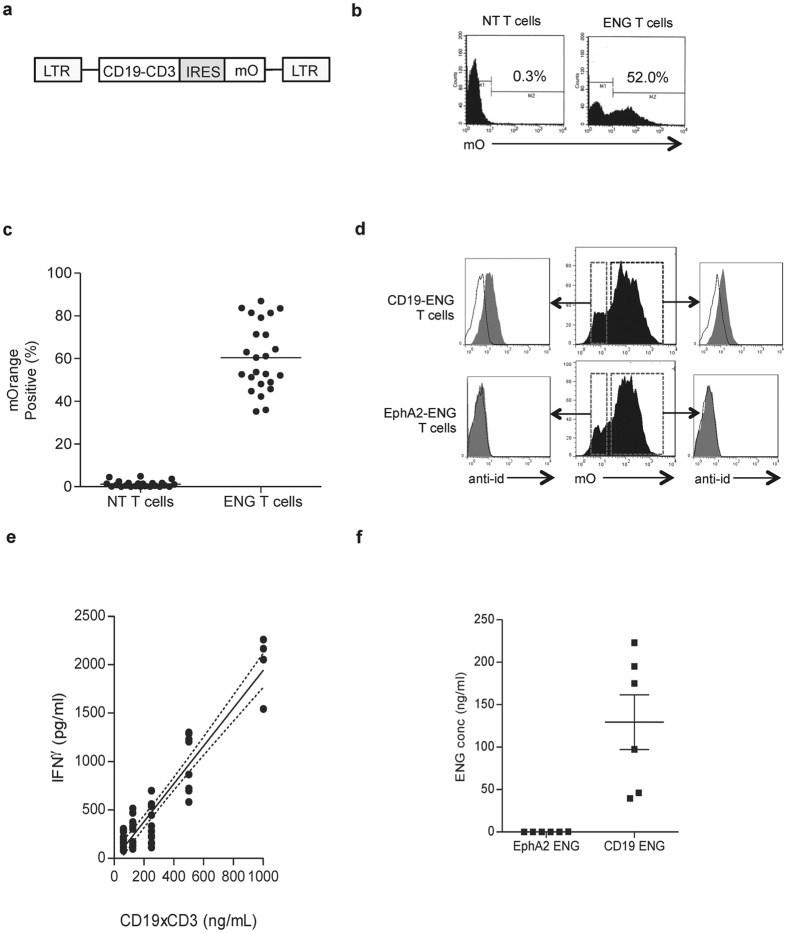
Generation of CD19-ENG T-cells. (**a**) Retroviral construct used to generate CD19 ENG T cells. (**b**,**c**) Five to 7 days after transduction, mOrange expression was measured by flow cytometry, and 60.4 ± 16% of the T-cells were mOrange positive (n = 24; range 35–87%; 95% CI 53.7–67.2). (**D**) A CD19-specific scFv Id antibody was used to detect CD19-ENG molecules. mOrange-positive and negative T cells stained positive (filled curve) for CD19-ENG in contrast to samples that were stained with secondary antibody alone (open curve). EphA2-ENG T cells were not stained by the CD19-specific scFv Id antibody, confirming specificity. (**e**,**f**) The concentration of CD19-ENG in media from CD19-ENG or EphA2-ENG T cells was determined using a coculture bioassay. (**e**) A standard curve was generated using recombinant CD19xCD3 protein. (**f**) CD19-ENG T-cell media had CD19-ENG protein concentrations ranging 39.7–223 ng/mL; (95%CI 46.6–212.3). No CD19-ENG molecules were detected in media of EphA2-ENG T cells.

T cells were transduced with a retroviral vector encoding a CD19-specific T-cell engager an IRES and mOrange (Fig. 1a). Five to 7 days after transduction, mOrange expression was measured by flow cytometry, and 60.4 ± 16% of the T cells were positive (n = 24; range 35–87%; 95% CI 53.7–67.2, Fig. 1b,c). Both mOrange-positive and -negative T cells had CD19-ENG molecules bound to their cell surface as judged by FACS analysis using a CD19-specific scFv Id antibody, indicating that CD19-ENG molecules are secreted from transduced cells and then bind to CD3 on the T-cell surface. T cells expressing an ENG molecule specific for an irrelevant antigen (EphA2; EphA2-ENG T cells) were not stained confirming specificity (Fig. 1d).

To quantify secretion of CD19-ENG molecules by CD19-ENG T cells, we used a coculture bioassay. While media of CD19-ENG T cells contained 129.4 ± 79.0 ng/ml CD19-ENG molecules (range 39.7–223 ng/mL; 95%CI 46.6–212.3), no CD19-ENG molecules were detected in media from T cells secreting an ENG specific for an irrelevant tumor antigen (EphA2-ENG T cells; Fig. 1e,f). Thus, our results demonstrate that it is feasible to generate T cells that secrete CD19-ENG molecules.

### CD19-ENG T cells recognize and kill CD19-positive target cells in antigen dependent manner

**Figure 2:**
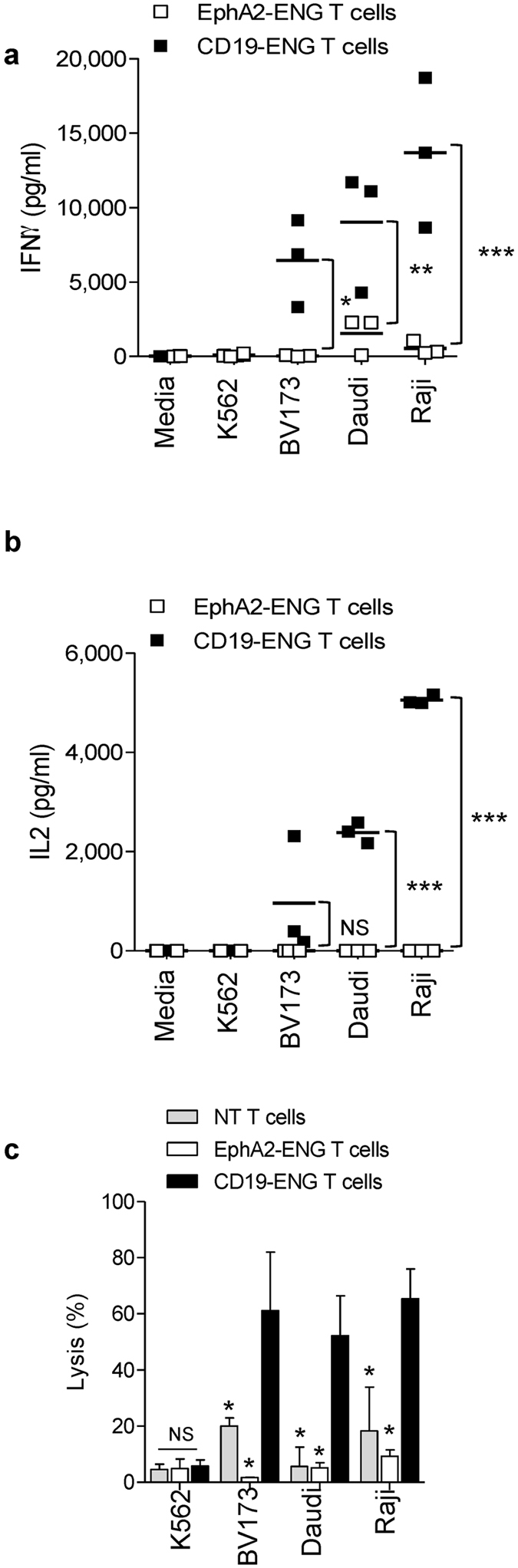
*In vitro* characterization of CD19-ENG T cells. (**a**,**b**) CD19-ENG or EphA2-ENG T cells were cocultured with CD19-positive (BV173, Daudi, Raji) or -negative (K562) tumor cells at a ratio of 2:1. After 48 hours, IFNγ and IL2 production was determined by ELISA (n = 3; assay performed in duplicates; *p < 0.05; **p < 0.01; ***p < 0.001; NS: not significant). (**c**) Cytotoxicity assays were performed using CD19-ENG, EphA2-ENG, and NT T cells as effectors and CD19-positive (BV173, Daudi, Raji) and negative (K562) tumor cells as targets at a E:T ratio of 2.5:1 (mean + SD; n = 3; assay was performed in duplicates; BV173, Raji, or Daudi: CD19-ENG vs EphA2-ENG T cells: *p < 0.001; CD19-ENG vs NT T cells: *p < 0.001; K562: CD19-ENG vs EphA2-ENG T cells: NS; CD19-ENG vs NT T cells: NS).

To evaluate if CD19-ENG T cells recognize CD19-positive target cells we performed coculture assays. CD19-ENG T cells secreted IFNγ after coculture with CD19-positive tumor cells (BV173, Daudi, Raji) while EphA2-ENG T cells did not (p < 0.001; Fig. 2a). Significant IL2 production was only observed in the presence of CD19-positive Raji and Daudi cells (Fig. 2b), which express co-stimulatory molecules such as CD80 and CD86, in contrast to BV173 cells (Supplementary Fig. 1). In cytotoxicity assays CD19-ENG T cells killed CD19-positive but not CD19-negative cells (Fig. 2c). NT and EphA2-ENG T cells had no cytolytic activity confirming the specificity of CD19-ENG T cells. These results indicate that CD19-ENG T cells recognize tumor cells in an antigen-dependent manner as judged by cytokine production and tumor killing.

### CD19-ENG T cells redirect bystander T cells to CD19-positive target cells

**Figure 3:**
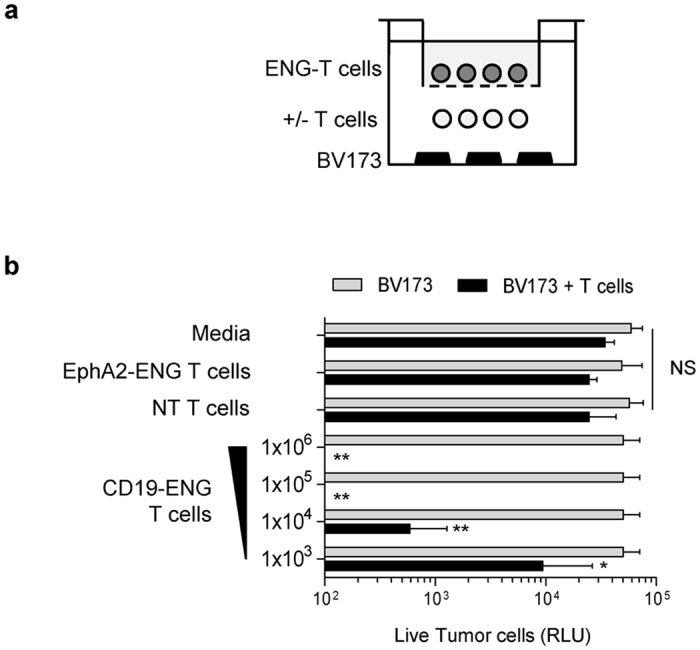
CD19-ENG T cells are able to recruit bystander T cells. (**a**) Schematic representation of transwell assay. (**b**) 1 × 10^6^ NT T-cells and 0.5 × 10^6^ BV173.ffLuc cells were plated in the bottom well and CD19-ENG T cells in the insert well. The number of plated CD19-ENG T cells ranged from 10^3^ to 10^6^. EphA2-ENG T cells or media in the insert well and bottom wells without NT T cells served as controls. After 48 hours, live BV173.ffLuc cells were determined by luciferase assay (n = 3; *p < 0.05; **p < 0.01).

To demonstrate that CD19-ENG T cells were able to recruit bystander T cells to CD19-positive targets we used transwell assays. BV173.ffLuc cells were plated with or without NT T cells in the bottom well, and ENG T cells or NT T cells were plated in the insert well (Fig. 3a). Tumor cell killing was dependent on the presence of NT T cells in the bottom well and CD19-ENG T cells in the insert well, indicating that T cells secrete ENG molecules to redirect NT T cells to CD19-positive targets (Fig. 3b). No cell killing was observed if EphA2-ENG or NT T cells were plated in insert wells, indicating that killing was antigen specific. To further demonstrate that bystander T-cell killing does not depend on T cells that produce the engager molecules, we expressed CD19-ENG or EphA2-ENG in NK cells, and performed cytotoxicity assays. CD19-ENG NK cells were able to induce BV173 killing in the presence of T cells, indicating that ENG-secreting cells that are not activated themselves by ENG molecules can redirect bystander T cells to tumor cells. Unmodified NK cells, or modified to secrete EphA2-ENG did not induce target cell killing (Supplementary Fig. 2). Thus, the results presented in this section demonstrate that ENG-secreting cells redirect bystander T cells to tumor cells in an antigen-dependent manner.

### CD19-ENG T cells have potent antitumor activity *in vivo*

**Figure 4:**
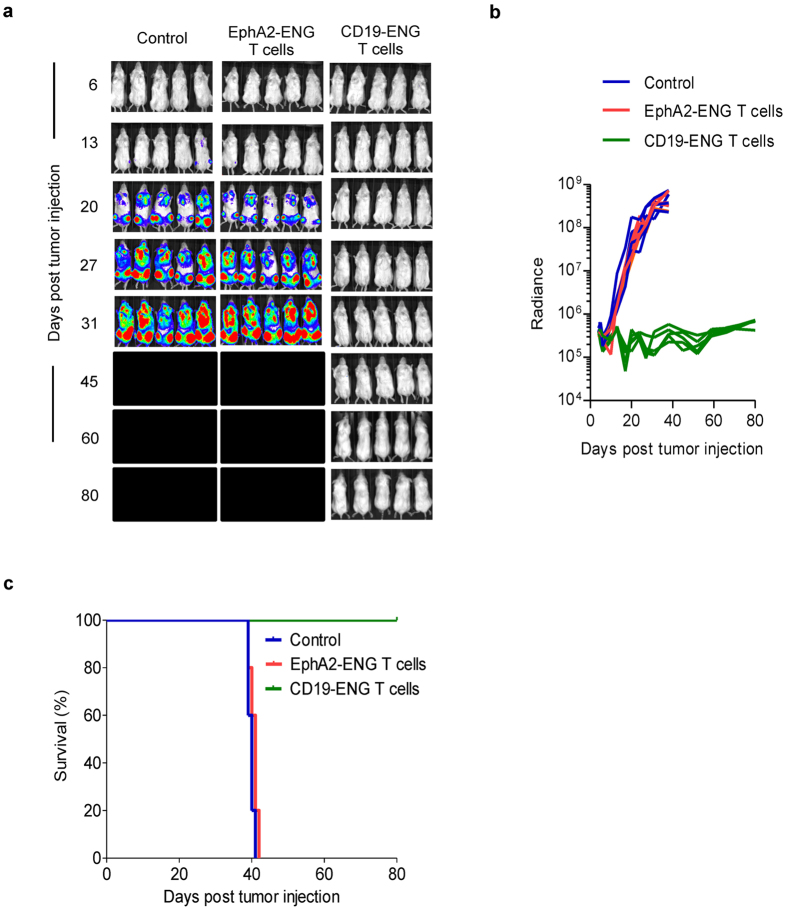
CD19-ENG T cells have potent antitumor activity *in vivo*. Antitumor activity of CD19-ENG T cells in the i.v. BV173 leukemia NSG xenograft model. Mice received an i.v. dose of 1 × 10^7^ CD19-ENG (n = 5) or EpHA2-ENG T cells (n = 5) and an i.p. dose of IL2 (1500 units) 7, 14, and 21 days after i.v. injection of 3 × 10^6^ BV173.ffLuc cells. Untreated animals served as controls (n = 5). Tumor growth was followed by bioluminescence imaging. (**a**) Images of animals. (**b**) Quantitative bioluminescence imaging results for each mice (radiance = photons/sec/cm^2^/sr) over time (p < 0.05 starting day 6 post 1^st^ T-cell injection for CD19-ENG vs EphA2-ENG T cells, and CD19-ENG T cells vs controls). (**c**) Kaplan-Meier survival curve (Control vs EphA2-ENG T cells: NS; Control vs CD19-ENG T cells: p = 0.0019; EphA2-ENG vs CD19-ENG T cells: p = 0.0021).

**Figure 5:**
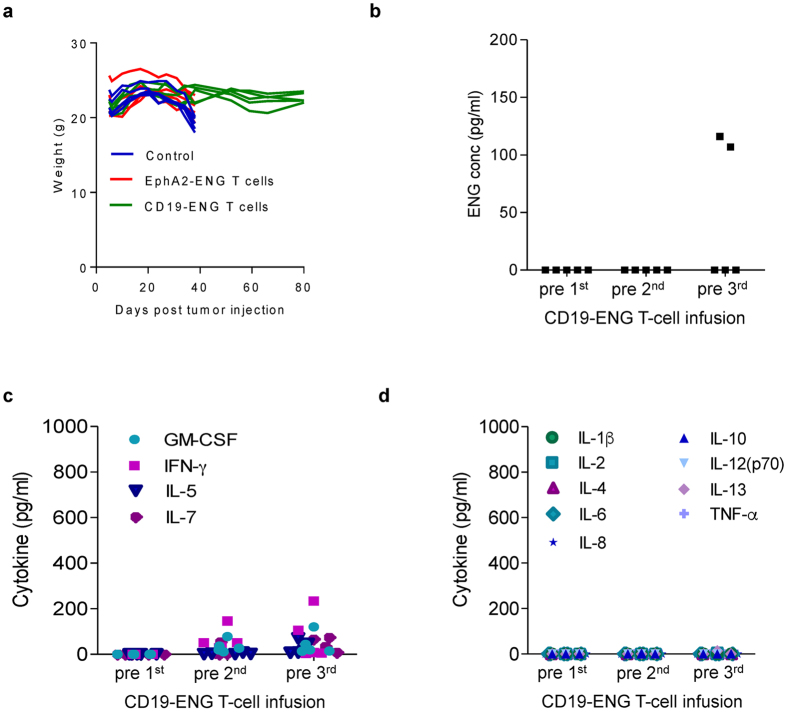
Safety profile of CD19-ENG T cells. (**a**) Untreated (Control) and mice treated with EphA2-ENG T cells started to lose weight and died before day 40, the weight of mice treated with CD19-ENG T cells remained stable throughout the experiment. (**b**) Before the 1^st^, 2^nd^ and 3^rd^ T-cell infusion blood from BV173 leukemia-bearing mice treated with CD19-ENG T cells (n = 5) was collected by retro-orbital bleeding and the concentration of ENG molecules was determined using our bioassay. Only two of 5 samples pre 3^rd^ T-cell infusion had detectable concentration of engager molecules in their blood. (**c**,**d**) At the same time points cytokines were determined using a Milliplex MAP High Sensitivity Human Cytokine Panel – Premixed 13 Plex (EMD Millipore, Billerica, MA) as per the manufacturer’s instructions. (**c**) GM-CSF, IFNγ, IL5, and IL7 were detectable at low levels (median: 19 pg/ml; range: 0–234 pg/ml) before the 2^nd^ and 3^rd^ infusion. (**D**) IL1b, IL2, IL4, IL6, IL8, IL10, IL12(p70), IL13, TNFα) were undetectable.

We evaluated the antitumor activity of CD19-ENG T cells in the i.v. BV173 leukemia NSG xenograft model. Mice received 1 × 10^7^ CD19-ENG T cells or EphA2-ENG T cells i.v. with one i.p. dose of 1500 IU IL2 on days 7, 14, and 21 post tumor cell injection. CD19-ENG T cells eradicated BV173 cells (Fig. 4a,b) resulting in disease-free survival of all mice in contrast to untreated mice and mice treated with EphA2-ENG T-cells (p < 0.05; Fig. 4c), which lost weight (Fig. 5a) and died within 40 days of BV173 injection.

To determine if leukemia control was achieved without prolonged, systemic exposure to ENG molecules we determined the concentration of CD19-ENG in the peripheral blood of mice at baseline and prior to the 2^nd^ and 3^rd^ infusion T-cell infusion. ENG molecules were only present at low levels (107 pg/ml, 116 pg/ml) in 2 out of 5 samples before the 3^rd^ T-cell infusion (Fig. 5b). To assess safety, we determined the presence of human cytokines in the peripheral blood of mice at the same time points. While we could detect low levels of GM-CSF, IFNγ, IL5, or IL7 prior to the 2^nd^ and 3^rd^ T-cell infusion (median: 19 pg/ml; range: 0–234 pg/ml), no increase of all other measured cytokines (IL1β, IL2, IL4, IL6, IL8, IL10, IL12(p70), IL13, TNFα) was observed (Fig. 5c,d).

**Figure 6:**
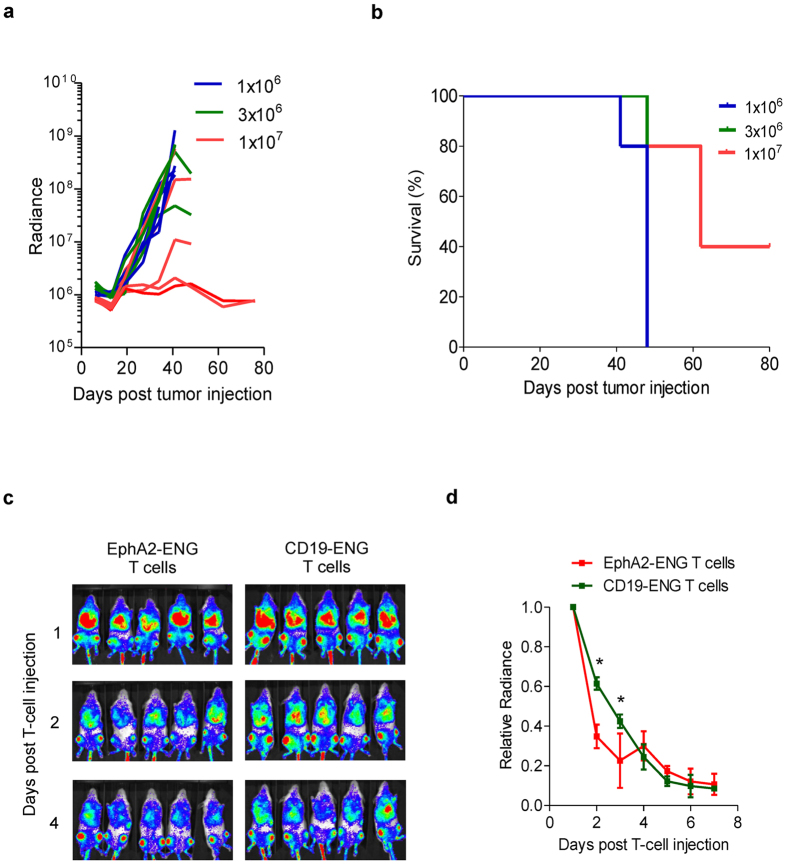
CD19-ENG T cells do not expand in the BV173 model *in vivo* and antitumor activity depends on T-cell dose. (**a**,**b**) Seven days after i.v. injection of 3 × 10^6^ BV173.ffLuc cells mice received a single i.v. dose of 1 × 10^6^, 3 × 10^6^, or 1 × 10^7^ CD19-ENG T cells (n = 5 mice per group). Tumor growth was followed by bioluminescence imaging. (**a**) Quantitative bioluminescence imaging results for each mice (radiance = photons/sec/cm^2^/sr). (**b**) Kaplan-Meier survival curve (1 × 10^7^ vs 3 × 10^6^ CD19-ENG T cells: p = 0.02; 1 × 10^7^ vs 1 × 10^6^ CD19-ENG T cells: p = 0.02; 3 × 10^6^ vs 1 × 10^6^ CD19-ENG T cells: p = NS). (**c**,**d**) eGFP.ffLuc expressing CD19-ENG or EphA2-ENG T cells were injected i.v. into NSG mice 7 days after i.v. BV173 tumor-cell injection (n = 5 mice per group). Day 2 and 3 post T-cell injection there was a significant difference (*p < 0.05) between both groups.

Having established that 3 doses of CD19-ENG T cells in conjunction with a single dose of IL2 can safely eradicate leukemia, we determined the minimal effective dose of CD19-ENG T cells without IL2. Mice were treated on day 7 with 1 × 10^6^, 3 × 10^6^ or 1 × 10^7^ CD19-ENG T cells. While 1 × 10^6^ or 3 × 10^6^ T cells were ineffective, 3 out of 5 mice treated with 1 × 10^7^ CD19-ENG T cells had a response with 2 being complete (Fig. 6a,b).

To investigate if the antitumor activity of CD19-ENG T cells depended on significant T-cell expansion we injected BV173 leukemia-bearing mice on day 7 with 1 × 10^7^ CD19-ENG or EphA2-ENG T cells that were genetically modified with a 2^nd^ retroviral vector encoding eGFP.ffLuc (Supplementary Fig. 3). While neither EphA2-ENG or CD19-ENG T cells expanded *in vivo* as judged by bioluminescence imaging (Fig. 6c,d), CD19-ENG T cells persisted longer resulting in a significant difference (p < 0.05) in bioluminescence imaging day 2 and 3 post T-cell injection in comparison to EphA2-ENG T cells.

**Figure 7:**
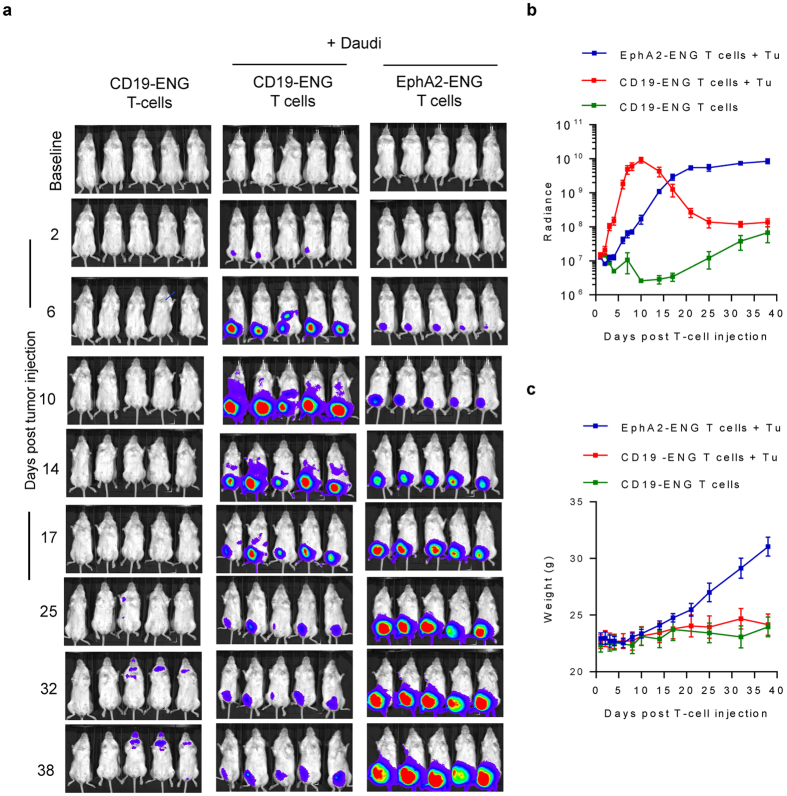
CD19-ENG T cells expand locally in the Daudi model *in vivo*. On day 14 post right lower quadrant I.P. injection of Daudi cells, mice received 3 × 10^6^ eGFP.ffLuc-expressing CD19-ENG or EphA2-ENG T cells. Non-tumor bearing mice that received 3 × 10^6^ eGFP.ffLuc-expressing CD19-ENG served as controls. (**a**) Images of animals. (**b**) Quantitative bioluminescence imaging results (radiance = photons/sec/cm^2^/sr; mean +/− SD is shown). CD19-ENG T cells + Daudi vs EphA2-ENG T cells + Daudi: p < 0.05 from day 3 to day 14; CD19-ENG T cells + Daudi vs CD19-ENG T cells: p < 0.05 starting day 3 until day 32. (**c**) Weight (mean +/− SD) for all three mice groups (EphA2-ENG T cells + Daudi vs CD19-ENG T cells or CD19-ENG T cells + Daudi: p < 0.05 starting day 25; CD19-ENG T cells + Daudi vs CD19-ENG T cells: p = NS).

To explore if CD19-ENG T cells home to tumor sites and expand in the presence of costimulatory molecules we used a local Daudi model in which tumor cells were injected I.P. in the right lower quadrant. On day 14 post tumor cell injection, mice received 3 × 10^6^ eGFP.ffLuc-expressing CD19-ENG or EphA2-ENG T cells. Non-tumor bearing mice receiving 3 × 10^6^ eGFP.ffLuc-expressing CD19-ENG T cells served as controls. CD19-ENG or EphA2-ENG T cells homed to tumor sites (Fig. 7a), and CD19-ENG T cells proliferated immediately in contrast to EphA2-ENG T cells as judged by bioluminescence imagining, indicative of antigen-dependent T-cell expansion (Fig. 7a,b). Starting day 14, there was a decline in bioluminescence signal of CD19-ENG T cells. CD19-ENG T-cell infused mice continued to grow with no significant change in weight in comparison to non-tumor bearing control mice (Fig. 7**c**). In contrast, EphA2-ENG T cells started to proliferate on day 6 post T-cell injection. While the bioluminescence signal continued to increase, mice developed visible tumors in the right lower quadrant, and ascites as judged by a significant weight gain (EphA2-ENG T cells + Daudi vs CD19-ENG T cells or EphA2-ENG T cells + Daudi: p < 0.05 starting day 25), necessitating euthanasia on day 39 post EphA2-ENG T-cell injection.

## Discussion

Here we describe the generation of CD19-ENG T cells and demonstrate that these cells recognize and kill CD19-positive target cells in an antigen-dependent manner, redirect ‘bystander’ T cells to CD19-positive target cells, and have potent antitumor activity *in vivo*.

Modifying T cells with CARs or αβ T-cell receptors (TCRs) is an attractive strategy to readily generate tumor-specific T cells *ex vivo*^28^,^29^, and early phase clinical studies have shown impressive clinical responses especially for CD19-positive hematological malignancies. However, these genetic modification strategies do not redirect resident, bystander T cells to tumor cells. We and others had previously shown than T cells can be genetically modified with diabodies or bispecific T-cell engagers to redirect modified T cells and bystander T cells to solid tumor antigens such EphA2 or CEA^20^,^21^. We now extend these findings to CD19 and demonstrate that T cells, which are genetically modified to secrete CD19-specific T-cell engagers and demonstrate their potent effector function *ex vivo* and *in vivo*.

T cells were readily transduced by our retroviral vector encoding the CD19-ENG molecule and mOrange as judged by mOrange expression. We confirmed the secretion of CD19-ENG molecules using a bioassay. While bioassays are routinely used to quantify the biological activity of cytokines^30^, we are planning to develop an ELISA in the future to directly measure the CD19-ENG molecule protein concentration. CD19-ENG T cells recognized CD19-positive target cells in coculture assays as judged by cytokine production and cytolytic activity. However, consistent IL2 production by CD19-ENG T cells was dependent on the presence of co-stimulatory molecules on the cell surface of target cells. Target-dependent IL2 production was also observed in our previous study with EphA2-ENG T cells, in which we observed *in vivo* T-cell expansion for the cell line that induced the highest amount of IL2 production of ENG T cells^21^. Indeed, while we did not observe *in vivo* expansion of CD19-ENG T cells in the BV173 model, we observed significant *in vivo* T-cell expansion in the Daudi model, in which the lymphoma cells express costimulatory molecules. Thus, robust IL2 production of ENG T cells after target cell encounter most likely predicts their ability to proliferate *in vivo*. Provision of costimulation could be achieved by expressing either costimulatory molecules such as CD80 or CD137L on the cell surface of T cells^31^ or a second engager molecule with a costimulatory domain^32^. Both strategies would provide costimulation to the genetically modified T cells, as well as to bystander T cells that are in close proximity.

CD19-ENG T cells had potent antitumor activity in the i.v. BV173 leukemia model and in the localized Daudi model. We confirmed the antitumor activity of CD19-ENG T cells in the systemic Daudi model in which NSG mice were injected i.v. with Daudi.ffLuc cells, and were treated i.v. with 3 doses of CD19-ENG or NT T cells (Supplementary Fig. 4). Side effects of CD19-targeted immunotherapies with CD19-CAR T cells or blinatumomab include fever, malaise, cytokine release syndrome (CRS), and neurotoxicity^14^. In all three models, CD19-ENG T cells infused mice did not lose weight, and for the BV173 model we also demonstrated that trough cytokine levels are not elevated; in addition, we could not detect circulating CD19-ENG molecules prior to each T-cell infusion. Clearly, a detailed time course of cytokine and ENG molecule concentration in the peripheral blood of infused mice is needed in the BV173 and Daudi model to confirm our findings in the future. However, it is important to note that significant side effects were not observed with CD19-CAR T cells and blinatumomab in preclinical animal models^33^,^34^, highlighting the inherent limitations of xenograft models for the study of human T cells. An immunocompetent animal model would most likely be more realistic for additional efficacy and safety studies of CD19-ENG T cells and to determine how these adoptively transferred cells interact with the resident immune system^8^,^33^,^35^,^36^,^37^. In this regard we have generated murine T cells expressing murine T-cell engagers based on the activating, murine CD3ε monoclonal antibody 2C11, but were unsuccessful to express sufficient amounts of T-cell engagers to induce antigen-dependent murine T-cell activation (Rodriguez-Cruz *et al*. unpublished observation).

In summary, our study shows that CD19-ENG T cells have potent antitumor effects, and have the unique ability to redirect resident T cells to CD19-positive hematological malignancies. Given the ability to introduce multiple genes into T cells^38^,^39^, it should be feasible to further increase the potency of ENG T cells by transgenic expression of cytokines or costimulatory molecules^31^,^39^,^40^, or increase their safety by introducing a suicide gene such as inducible caspase 9 41. Thus, ENG T cells may be a promising addition to current CD19-targeted immunotherapy approaches that either rely on the continuous infusion of recombinant protein or the adoptive transfer of CAR T cells.

## Additional Information

**How to cite this article**: Velasquez, M. P. *et al*. T cells expressing CD19-specific Engager Molecules for the Immunotherapy of CD19-positive Malignancies. *Sci. Rep.* **6**, 27130; doi: 10.1038/srep27130 (2016).

## Supplementary Material

Supplementary Information
