# Supplementary material for: T cells expressing CD19-specific Engager Molecules for the Immunotherapy of CD19-positive Malignancies
Source: Sci Rep. 2016 Jun 3;6:27130. doi: 10.1038/srep27130 (PMC4891739; doi:10.1038/srep27130)
Supplement: Supplementary Information [file srep27130-s1.pdf]

T cells expressing CD19-specific Engager Molecules for the Immunotherapy of CD19-positive Malignancies

Mireya Paulina Velasquez, David Torres, Kota Iwahori, Sunitha Kakarla, Caroline Arber, Tania Rodriguez-Cruz, Arpad Szoor, Challice L. Bonifant, Claudia Gerken, Laurence JN Cooper, Xiao-Tong Song, Stephen Gottschalk

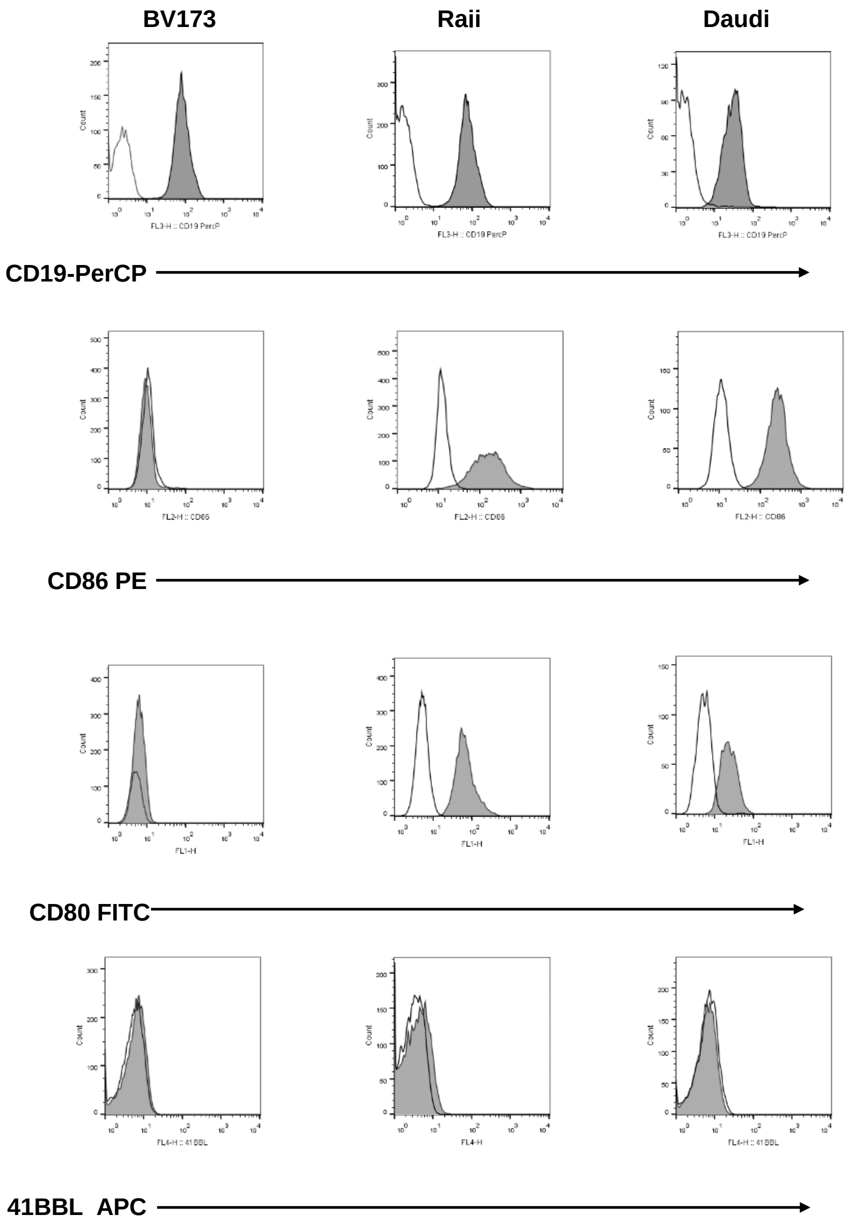

Supplementary Figure 1: Characterization of tumor cell lines. (A) BV173, Raji and Daudi tumor cell lines were stained for surface CD19 (PerCP), CD86 (PE), CD80 (FITC) and 41BBL (APC). All cell lines were positive for surface CD19 antigen. Only Daudi and Raji were positive for CD86 and CD80. None of the 3 cell lines expressed 41BBL (Isotype: black line, Antibody staining: shaded area)

Supplementary Figure 1

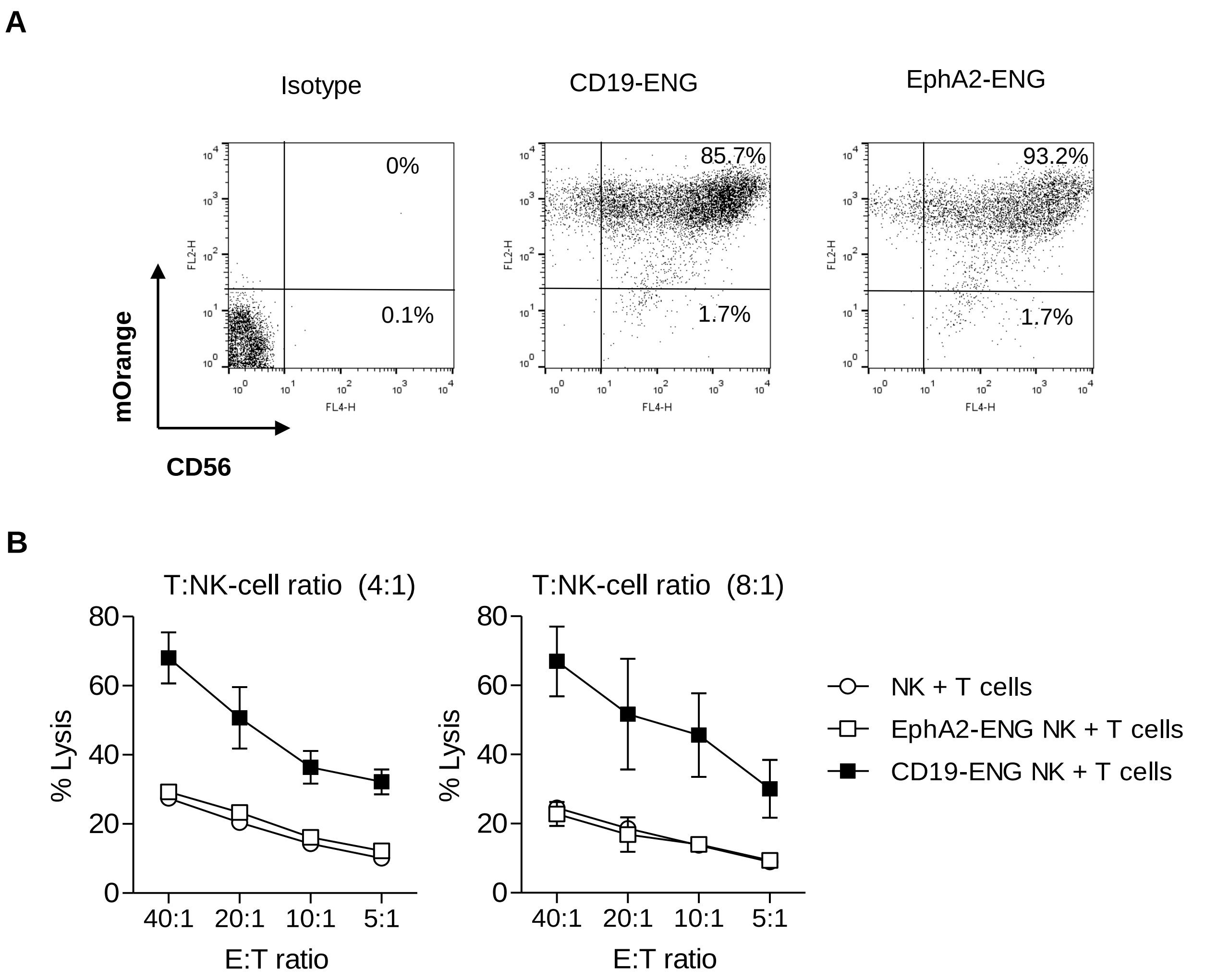

Supplementary Figure 2: NK cells secreting CD19-ENG are able to redirect bystander T cells to target cells. To demonstrate that bystander T cells can be activated by 'none T cells' secreting engagers, we generated CD19-ENG secreting NK cells. Briefly, NK cells were activated and expanded with irradiated K562 expressing 41BBL and membrane bound IL15 in the presence of IL2 as previously described.[1,2] On day 4post activation, NK cells were transduced on retronectin-coated plates with RD114-pseudotyped retroviral vectors. Before performing cytotoxicity assays, NK cells were rested for 2 days in media without cytokines. (**A**) Transduction efficiency of NK cells. (**B**) The ability of genetically-modified NK cells to redirect bystander T cells was evaluated in cytotoxicity assays. BV173 (CD19+) were used as target cells, and as effectors an admixture of nontransduced T cells and NK cells (ratio of 4:1 or 8:1). Only CD19-ENG NK cells were able to induce killing of BV173 cells by T cells (assay were preformed in triplicates; CD19-ENG NK cells + T cells vs EphA2-ENG NK cells + T cells: $p < 0.0001$; CD19-ENG NK cells + T cells vs NK cells + T cells: $p<0.0001$).

1 Fujisaki, H. *et al.* Expansion of highly cytotoxic human natural killer cells for cancer cell therapy. *Cancer Res.* **69**, 4010-4017 (2009).

2 Lapteva, N. *et al.* Large-scale ex vivo expansion and characterization of natural killer cells for clinical applications. *Cytotherapy.* **14**, 1131-1143 (2012).

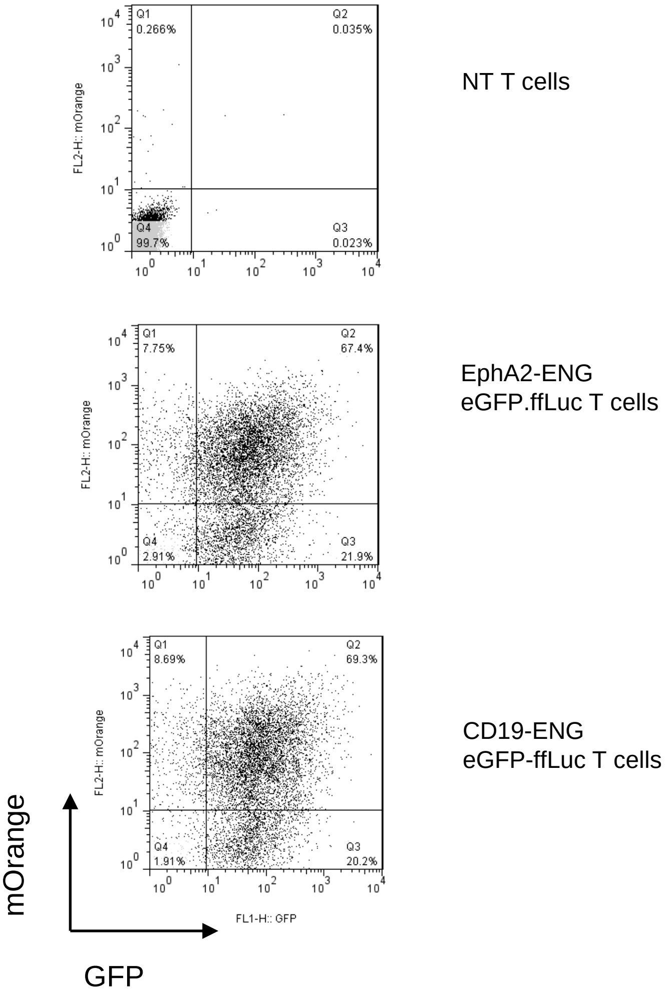

Supplementary Figure 3: FACS analysis of GFP.ffLuc and Engager expressing T cells. CD3/CD28-activated T cells were transduced with RD114-pseudotyped retroviral particles encoding CD19-ENG and mOrange, and eGFP.ffLuc. FACS analysis for mOrange and GFP.

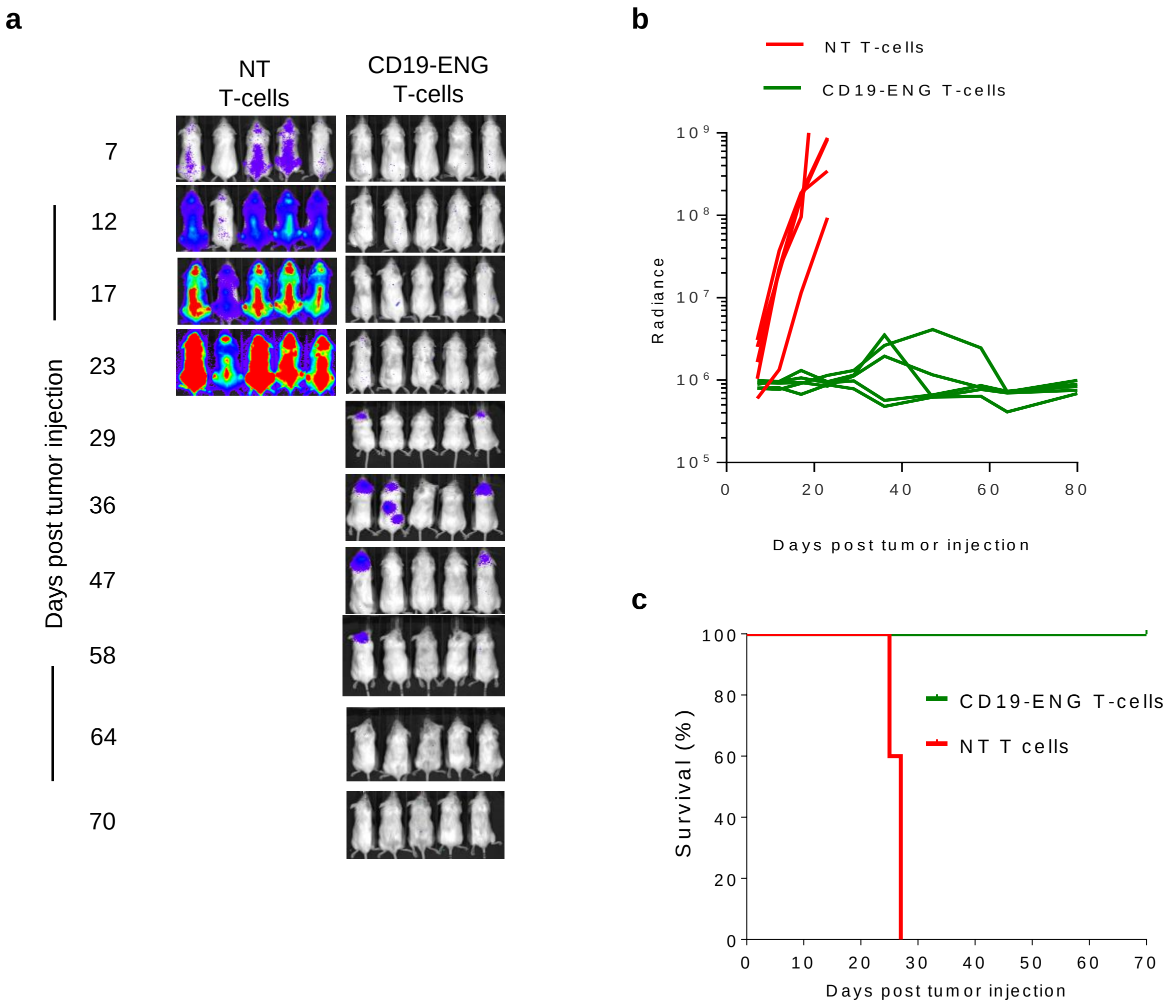

Supplementary Figure 4: Antitumor activity of CD19-ENG T cells in Daudi model. NSG mice were injected i.v. with $3x10^6$ Daudi.ffLuc cells. Three, 6, and 9 days after tumor cell injection, mice were treated i.v. with $1x10^7$ CD19-ENG or NT T cells. (**a**) Images of animals. (**b**) Quantitative bioluminescence imaging results for each mice (radiance=photons/sec/cm^2/sr) over time ($p<0.05$ starting day 14 after the 1st T-cell injection for CD19-ENG vs NT T cells). (**c**) Kaplan-Meier survival curve (CD19-ENG vs NT T cells: $p=0.0012$).

Supplementary Figure 4
